# Cumulative Exposure to Unconventional Natural Gas Development and the Risk of Childhood Cancer: A Registry-Based Case–Control Study

**DOI:** 10.3390/ijerph22010068

**Published:** 2025-01-07

**Authors:** Evelyn O. Talbott, Vincent C. Arena, Renwei Wang, Fan Wu, Natalie Price, Jeanine M. Buchanich, Caroline A. Hoffman, Todd Bear, Maureen Lichtveld, Jian Min Yuan

**Affiliations:** 1 Department of Epidemiology, School of Public Health, University of Pittsburgh, Pittsburgh, PA 15261, USA; faw19@pitt.edu (F.W.); nfp9@pitt.edu (N.P.); cah293@pitt.edu (C.A.H.); yuanj@upmc.edu (J.M.Y.); 2 Department of Biostatistics and Health Data Science, School of Public Health, University of Pittsburgh, Pittsburgh, PA 15261, USA; arena@pitt.edu (V.C.A.); jeanine@pitt.edu (J.M.B.); 3 UPMC (University of Pittsburgh Medical Center) Hillman Cancer Center, University of Pittsburgh, Pittsburgh, PA 15232, USA; wangr2@upmc.edu; 4 Department of Behavioral and Community Health Sciences, School of Public Health, University of Pittsburgh, Pittsburgh, PA 15261, USA; tobst2@pitt.edu; 5 Department of Environmental and Occupational Health, School of Public Health, University of Pittsburgh, Pittsburgh, PA 15261, USA; mlichtve@pitt.edu

**Keywords:** hydraulic fracturing, unconventional natural gas development (UNGD), childhood cancer, inverse distance weighting (IDW)

## Abstract

The rapid growth of unconventional natural gas development (UNGD), also known as hydraulic fracturing, has raised concerns of potential exposures to hazardous chemicals. Few studies have examined the risk of childhood cancer from exposure to UNGD. A case–control study included 498 children diagnosed with leukemia, lymphoma, central nervous system neoplasms, and malignant bone tumors during the period 2010–2019 identified through the Pennsylvania Cancer Registry. Cases were matched to controls using Pennsylvania birth records. For each subject, a new overall UNGD exposure metric was calculated which incorporates both spatial (proximity) and temporal (duration) aspects of well activity. Conditional logistic regression models were used to estimate the risk of combined and individual cancers by overall UNGD exposure, and well proximity. Children with a higher overall UNGD exposure (3rd/4th quartiles) had an increased risk for the four malignancies combined [OR] 1.69 (95% CI 1.01, 2.82) and 1.79 (95% CI 1.00, 3.19) compared to non-exposed children. Overall, individuals living within 0.5 miles of a UNGD site were 3.94 times (95% CI 1.66, 9.30) more likely to develop a malignancy compared to non-exposed children and the risk of lymphoma within 0.5 miles and 0.5–1 miles was also elevated [ORs of 5.05 (95% CI 1.09, 23.39) and 7.71 (95% CI 1.01, 59.00), respectively] compared to non-exposed. Our study found that overall UNGD cumulative activity as well as a proximity to UNGD wells were associated with an increased risk of childhood lymphoma and overall childhood cancers combined.

## 1. Introduction

Cancer in children and adolescents is the leading cause of death by disease past infancy among them in the United States [1]. A recent study by Siegel et al. [2] evaluated age-adjusted cancer incidence rates and trends of cancer among children and adolescents between 2003–2019. Overall cancer incidence rates were the highest for leukemia (46.6 per 1 million), brain and central nervous system (CNS) neoplasms (30.8), and lymphomas (27.3). Between 2003 and 2019, rates of leukemia, lymphoma, bone malignant tumors, and thyroid carcinomas increased, while melanoma rates decreased.

Many studies suggest environmental contaminants may play a role in the development of childhood cancers. These investigations have focused on pesticides and solvents, such as benzene, and most recently hazardous air pollutants including motor vehicle exhausts and environmental tobacco smoke [3]. Associations have been noted for CNS neoplasms, refs. [4,5,6] leukemia, refs. [4,5,6,7,8,9,10,11] and lymphoma [4,5,6]. Exposures to diagnostic medical radiation such as computed tomography (CT) and X-rays during childhood and/or the mother’s exposure to radiation during pregnancy have been found to be associated with a slight increase in risk of leukemia and brain tumors, and possibly other cancers [12]. Additionally, limited research indicates that pesticides may be a potential risk factor for Ewing’s sarcoma in children [6,7].

Hydraulic fracturing (also known as fracking) is a type of unconventional natural gas development (UNGD) used to extract natural gas from underground shale rock formations. UNGD has been rapidly expanding in Pennsylvania since 2005 [13,14] with a particularly high density in Southwestern Pennsylvania. In 2005, only 13 permits were issued, and two wells were completed in Pennsylvania [13]. The number of active wells increased to approximately 11,500 in 2022 [15]. This UNGD process consists of four phases: preparation, drilling, hydraulic fracturing, and production [16]. To extract the gas, fracturing fluid is injected into the rock which “fractures” and releases the trapped gas [16].

This relatively new drilling technique has raised concerns about the potential exposure to multiple hazardous substances released into the environment. Fracturing fluid typically consists of over 1 million gallons of water, sand, and chemical additives per well. A number of these chemicals are known and suspected carcinogens [17]. Previous systematic exposure assessments by Xu et al. [18] and Elliott et al. [19] investigated both air and water pollutants from fracturing according to the International Agency for Research on Cancer (IARC)’s Classification of carcinogenicity [20]. The studies reported the presence of known human carcinogens in the UNGD-related pollutants including 1,3-butadiene, benzene, cadmium, ethanol, ethylene oxide, formaldehyde, quartz, and heavy metals, as well as naturally occurring uranium and radium. Exposure to these human carcinogens may cause alterations in genes that lead to uncontrolled cell growth and eventually cancer [1]. Unconventional natural gas development releases not only fossil fuel emissions in well preparation but the aftermath of production creates a large amount of wastewater (flowback) that must be disposed of.

There have been three studies that examined the associations between UNGD activity and risk of childhood cancer. Fryzek et al. [21] conducted an ecological investigation of childhood cancer incidence rates at the county level within Pennsylvania and found no significant increase in the incidence rates of the total cancer or leukemia in post- versus pre-UNGD years, but a slight increase in CNS neoplasms in years after the UNGD activities began. This investigation was followed by two case–control studies of childhood cancer, one in Colorado [22] and the other [23] in Pennsylvania. Both used residential proximity to active UNGD sites and inverse-distance weighted (IDW) well counts as surrogates of exposure. The study by McKenzie et al. [22] included children and adolescents (0–24 years of age) with acute lymphoblastic leukemia (ALL, n = 87) and non-Hodgkin lymphoma (NHL, n = 50) as cases and 528 children with cancers other than hematologic malignancies as controls in rural Colorado during 2001–2013. Overall, the study did not find an association of the risk of these malignancies with the annual IDW well count within 16.1 km (i.e., 10 miles [mi]) radius of residence. However, for ages 5–24, children with ALL were 4.3 times as likely to live in the highest tertile of the IDW well count compared those living more than 16.1 km (about 10 mi) away from a UNGD well (95% CI: 1.1 to 16), with an increase in risk across tertiles of well counts (P trend = 0.035). Clarke et al.’s study [23] included 405 children (2–7 years of age) diagnosed with ALL in Pennsylvania during 2009–2017 and 2080 control children randomly chosen from the Pennsylvania birth records that were matched by birth year, but not sex, race, or county. The study found an elevated odds (OR = 1.98, 95% CI 1.06–3.69) of children with ALL whose residences at birth were located within 2 km (1.2 miles) of any UNGD wells during the primary window of exposure time studied (from 3 months prior to conception to 1 year prior to ALL diagnosis) compared with those living >2 km (>1.2 miles) away. With the caveats of these prior epidemiological studies in their study design and assessment of exposures to UNGD activities, the findings of these studies suggest a potential link between UNGD and the risk of hematologic malignancies and CNS neoplasms.

Given the increased UNGD proliferation within Southwestern Pennsylvania since 2005 and health concerns of local communities, we conducted a case–control study assessing the association for the overall activities and residential proximity of UNGD with the risk of common childhood cancer types including leukemia, lymphoma, and CNS neoplasm. These are also the types of cancers found to be associated with various environmental exposures including hydraulic fracturing in both adults and children in the literature [1]. Our study also included malignant bone tumors due to community concerns about a potential link for UNGD to Ewing’s family of tumors in Southwestern Pennsylvania [24,25]. In addition to the overall activities across the four phases of UNGD process and the residential proximity to UNGD sites for each residence per study subject, we assessed other potential environmental exposures including Superfund, Toxic Release Inventory (TRI), and Uranium Mill Tailings Remediation (UMTRA) sites as independent variables or covariates with the risk of childhood cancer.

## 2. Materials and Methods

We employed a matched case–control study design for the present study. Both Internal Review Boards (IRBs) of the University of Pittsburgh (STUDY21020141) and the Department of Health of Commonwealth of Pennsylvania (PADOH) approved the present study.

### 2.1. Study Population

#### 2.1.1. Cancer Cases

All cancer cases were identified by the Pennsylvania Cancer Registry, a part of the National Program of Cancer Registries (NPCR) administered by the Centers for Disease Control and Prevention (CDC). Eligibility criteria were as follows: Cancer types included leukemia, lymphoma, CNS neoplasms, and malignant bone tumors defined by the International Classification of Childhood Cancer Recoded Third Edition ICD-O-3/IARC 2017 (see details in Table S1). Cases with leukemia, lymphoma, and CNS malignant neoplasms were diagnosed at 19 years of age or younger during 2010–2019. Children with Ewing’s family of tumors who were diagnosed at 20–29 years of age were included in the study to increase the sample size due to the disease’s extreme rarity. Both the residences at the time of birth and at cancer diagnosis were from one of the following 8 counties in Southwestern Pennsylvania: Allegheny, Armstrong, Beaver, Butler, Fayette, Greene, Washington, and Westmoreland. Out of a total of 593 cases ascertained from the PA Cancer registry in the original dataset for the period 2010 through 2019, and after the application of further eligibility criteria cited above, the following cases were removed as follows:

- A total of 41 were ineligible cancer cases based on the Third Edition ICD-O-3/IARC 2017).
- A total of 20 were born outside of the eight-county study area.
- A total of 25 were diagnosed within the city of Pittsburgh with no fracturing. Cases with the residence at birth or cancer diagnosis within the city of Pittsburgh in Allegheny County were excluded from the present study due to a city’s ordinance against fracking. In total, 507 cases were eligible for the study.

#### 2.1.2. Control Subjects

The source for control subjects was birth records from the Pennsylvania Bureau of Health Statistics and Registries within the PADOH from 1990 through 2019. For each case, we randomly selected one control subject who was matched to the index case by date of birth (±45 days), the same race (white, black, and other), sex, and county of residence. If a control subject was matched to multiple cases or multiple control subjects to one case, a simple random sampling algorithm without replacement was used to determine the matched case–control pair. Nine case/control pairs were excluded due to a mismatching on the county of residence or race, resulting from data entry errors. A total of 498 case–control matched pairs were available for the study.

### 2.2. Measurement of Exposure to UNGD

The measurements of exposure to UNGD activity were estimated using official open-source electronic datasets from the Pennsylvania Department of Environmental Protection (PADEP) [26] and the Department of Conservation and Natural Resources [27]. These source activity datasets contained the coordinates of individual UNGD well locations and the start and end dates for each of the four phases of UNGD well processes. Efforts were made, as best as possible, to independently verify and correct missing and/or inconsistent data elements. These source datasets serve as the basis for when well sites were active. Any well that was active or completed during the defined period of exposure (i.e., birth till the date of diagnosis of index cases is counted toward the exposure metric). Any well that was active after the time of diagnosis was censored. The mean duration for the four UNGD process phases were as follows: 30 days for well pad preparation, 145 days for drilling, 20 days for hydraulic fracturing, and 2239 days for production. 

*Two different exposure* metrics were created as follows: an overall UNGD exposure measure, and a proximity measure. There are several previous papers related to UNGD activity and birth/cancer outcomes using an inverse-distance weighted index of UNGD activity [22,28]. We geocoded the residential address at birth for each of the study subjects using ArcGIS 10.6. This birth address was used for the calculation of the duration of exposure for cases and controls.

The *cumulative exposure measure* or *overall UNGD exposure* metric was created to estimate the length of the exposure from any activity of wells within a 5 mile buffer from the residence at birth. This *cumulative exposure measure* was calculated separately for two predefined windows of time: *pregnancy time period* was defined as the period of time prior to birth as derived from gestational age in weeks on the birth certificate; *birth to diagnosis time period* was defined as the date of birth until the cancer diagnosis date (for cases) or index date (for controls). Only those days of well activity that coincided with the exposure window (e.g., pregnancy time period or birth to diagnosis) were included in the calculation.

The *overall UNGD exposure* metric captures the length of exposure to well activity while simultaneously adjusting for the distance from the birth residence. Thus, there is less contribution from wells with greater distances than wells with shorter distances to the *overall UNGD exposure*.

For each well, the Euclidean distance between it and the residence at birth was calculated. If the distance was within 5 miles, the number of days of well activity was calculated and then weighted by the inverse of the squared Euclidian distance for that site. The *overall UNGD exposure* for each individual is estimated as the sum of the contributions from all wells for each case and control within five miles from their birth residence until their index cases’ time of diagnosis. If the sum is zero, this individual was classified as non-exposed.

The second exposure metric was the *closest well proximity* metric for each person. A Euclidean distance from the residence at birth to each individual UNGD well was calculated and the minimum value selected. Individuals whose closest well proximity exceeds 5 miles were considered non-exposed. Similar to the overall UNGD exposure metric, the *closest well proximity* metric was calculated separately for the two predefined windows of time (*pregnancy time period* and *birth to diagnosis time period*). Only those wells active during the time window were used in the respective estimates.

For the purposes of analysis, the *closest proximity measures* were grouped into categories of pre-specified buffer zones: [0–0.5], (0.5–1], (1–2], and (2–5] miles [the “(” in front of a number denoted that the number was not included in the group category]. In addition, study subjects whose *closest proximity measures* were within 5 miles of any UNGD well(s) were classified as exposed, or otherwise they were considered non-exposed. The *overall UNGD exposure* metric was grouped into tertile or quartiles.

Given that UNGD did not begin until 2005 in Southwestern Pennsylvania and the age criteria for this study was 0–29 years at diagnosis from 2010 to 2019, there were 264 (52%) of the 498 cases and matched controls born before 2004 who did not have any exposure to UNGD during their pregnancy. This significantly limited the number of children for the analysis of this exposure time period. Therefore, our primary focus was the birth to diagnosis time period.

### 2.3. Other Covariates

The maternal age at childbirth, maternal education level, maternal smoking status, gestational age in weeks at birth, and birthweight were derived from the birth records. These variables were included in statistical modelling to account for their potential confounding effect on the UNGD-cancer risk association.

### 2.4. Other Environmental Exposures

In addition to the UNGD activity metrics, there were three environmental exposures sources included in our analysis: UMTRA sites, TRI sites, and Superfund sites. We used the same method to separately estimate the *closest proximity distance* metric from the residence at birth to TRI sites within a set of determined distance zones, a circular zone surrounding a subject’s residence (0.5, 1, 2, and 5 miles) for each study subject [29,30,31]. As for Superfund and UMTRA sites, of which there were fewer (see Figures S1 and S2), the buffer zone was living within 5 miles from these sites from birth to diagnosis (index) date. UMTRA sites are stationary legacy sites which have been identified and monitored by US agencies since the mid 1970’s. There were 235 TRI sites in the eight-county study area representing over 650 hazardous compounds monitored by the US Environmental Protection Agency (EPA) [29]. It should be noted that TRI data is self-reported by the company and is an estimate of the pounds of a compound of toxic materials released into the environment. It may not reflect a true amount released into the environment and thus can contribute to exposure misclassification.

Geographic locations of these stationary environmental exposures are shown in maps (Figures S1–S3). Four locations of UMTRA are in Figure S1. Mill tailings are defined as the sandy waste material from a conventional uranium mill. Milling is the first step in making fuel for nuclear reactors from natural uranium ore. UMTRA sites are areas designated by the US Department of Energy who monitor the clean-up of these mills and prevent the further contamination of ground water [30].

TRI sites are known facilities throughout the US that must report toxic chemical releases to the EPA yearly. Figure S3 shows the location of the TRI sites in the study area and surrounding counties regulated by the EPA. For the present analysis, we downloaded the 2015 data on all TRI inventory sites for the eight-county study area and all surrounding counties [29]. The year 2015 was chosen as a representative time-point based on the midpoint of the diagnosis time (i.e., 2010–2019) of cancer cases included in the study. Figure S2 shows Superfund sites, which is an environmental remediation program established by the EPA. The program is designed to investigate and clean-up sites contaminated with hazardous substances and includes seven sites within the eight-county study area [31].

### 2.5. Statistical Analysis

All the data analysis was conducted using SAS version 9.4 (SAS Institute, Cary, NC, USA). Descriptive statistics were computed and assessed for all outcomes and exposure measures, covariates, and characteristics of the 498 childhood cancer cases and their matched controls. For continuous variables, mean/standard deviation and median/inter quartile ranges were used; for categorical variables, frequency/percentiles were used. Chi-square testing assessed differences in percentages for sociodemographic and maternal characteristics between groups (e.g., cases vs. controls) when categorical; *t*-tests evaluated differences in means between groups when continuous. When appropriate, nonparametric tests were used.

The study’s main aim was to examine the association between the exposure to UNGD activity and childhood cancer risk. As such, conditional logistic regression modeling was used to assess this relationship. Separate regression models were used to estimate the relative risk (ORs and the 95% CIs) for all cancers combined (i.e., leukemia, lymphoma, CNS neoplasms, and malignant bone tumors) evaluating exposure metrics as exposed/unexposed, overall UNGD exposure, and closest well proximity as indicated by the buffer zone. Regression analyses were performed with and without adjustment for additional covariates. Multivariable-adjusted models included the following covariates: maternal age at childbirth (continuous), maternal education level (≤8th grade, high school, some college, or college degree or higher), maternal smoking status at childbirth (yes/no), gestational age (continuous in weeks), birthweight (continuous in grams), TRI (delineated as non-exposed or exposed within 5 miles), and UMTRA (non-exposed or exposed within 5 miles), as well as Superfund sites (non-exposed or exposed within 5 miles).

Similar logistic regression modeling was also performed for each of the four individual cancer types. Although this might have led to some analyses being underpowered, our study team, a priori, believed it was important to separately examine them due to their different biological characteristics. For the Ewing family of tumors (n = 20), unconditional logistic regression modeling to increase the sample size was performed separately from other malignant tumors of the bone by including all 498 controls with adjustments for matching variables (i.e., age at diagnosis, sex, race/ethnicity, and county of residence).

Significance testing was performed for individual ORs, as well as for the evaluation of the linear trend of increasing levels of UNGD activities using an ordinal variable (i.e., 0 for non-exposed and 1, 2, and 3 for tertiles or 1, 2, 3, and 4 for quartiles) with the risk of disease of interest. Similar logistic models were used for the decreasing buffer zone {non-exposed, (2–5] miles, (1–2] miles, (0.5–1.0] miles, and [0–0.5] miles} with the risk of disease of interest.

Additional conditional logistic regression modeling was performed to assess the relationship between the closest proximity to TRI sites (non-exposed, [0–0.5], (0.5–1], (1–2], and (2–5] miles) and all the cancers combined, adjusted for the covariates of maternal age at childbirth, maternal education level, maternal smoking status at childbirth, gestation age, and birthweight. This was repeated for individual cancers as well. We also constructed separate logistic models for UMTRA (non-exposed or exposed within 5 miles) and for Superfund sites (non-exposed or exposed within 5 miles).

## 3. Results

The distribution of the 498 cancer cases by primary site and other characteristics of children with newly diagnosed childhood malignancies excluding metastatic and secondary cancers are shown in Table 1. CNS and miscellaneous intracranial and spinal neoplasms comprised the largest group (38.8%), followed by leukemia and myeloproliferative diseases (31.5%), lymphomas (21.1%), and malignant bone tumors (8.6%) including 20 cases of Ewing family tumors (4.0%). The number of cases by year at diagnosis has a slightly downward trend. The distribution of ages 0–19 among all childhood cancer cases was similar to the total study population. Three cases diagnosed with Ewing family of tumors at 20–29 years were included in the study to maximize the sample size. They were also similar in distribution to the national data recorded by the NCI SEER Program [32].

Childhood cancer cases and their matched controls were 56.6% male, and 96.4% white. (Table 2). Fewer cases were included in the 1990–1994 birth cohort as some of the children “aged out” (i.e., were older than 19 years for the period of cancer diagnosis from 2010–2019). Allegheny County, being the most populated area, contributed 201 (40.4%) out of all the 498 childhood cancer cases to the study, followed by Westmoreland (17.5%), Butler (13.7%), and Washington county (12.0%). Mothers of cases and their matched controls reported similar levels of education (37.4% vs. 38.8% college graduates or higher) and non-smokers (79.7% vs. 81.9%). The average gestational age was 38 weeks for both case and control groups. The frequencies of normal birthweight (2501–4000 g) were comparable in cases (82.5%) and controls (85.5%).

### 3.1. Exposure to UNGD Activity and the Risk of Childhood Cancer During the Birth to Diagnosis Time Period

Table 3 shows the results for childhood lymphoma, leukemia, and CNS neoplasms combined and separate. Compared with the non-exposed group, children living within 5 miles of any UNGD wells had a non-significant 24% increased risk of the four malignancies combined. Subjects with a higher overall UNGD exposure had an increased risk of childhood malignancies combined for the third (OR = 1.69, 95% CI 1.01–2.81) and fourth quartiles (OR = 1.79, 95% CI 1.00–3.29) compared to non-exposed. Of the 498 case–control pairs, children diagnosed with any of the four malignancies combined were about four times more likely to live within 0.5 miles of a UNGD site than controls (OR = 3.94, 95% CI 1.66–9.30) compared to non-exposed. There is a statistically significant linear trend for closer proximity and the risk of childhood malignancy (*p* = 0.004). Among the 105 lymphoma cases and their matched controls, an exposure to UNGD activity from birth to the date of diagnosis was associated with an increased risk of lymphoma (OR = 2.24, 95% CI 0.92–5.47, *p* = 0.076). Children diagnosed with lymphoma were 5.68 (95% CI 1.58–20.48) and 3.96 (95% CI 1.01–15.49) more likely to be in the third and fourth highest quartile, respectively, of the overall UNGD exposure within 5 miles than their matched controls. Furthermore, children who lived within 2–5, 1–2, 0.5–1, and <0.5 miles from any UNGD site were at increased risk of lymphoma, ORs (95% CIs): OR = 2.06 (0.83–5.13), 2.45 (0.77, 7.83), OR = 5.05 95% CI (1.09–23.39), and OR= 7.71, 95% CI (1.01–59.00), respectively, compared with the non-exposed group (*p* value for trend = 0.015). For leukemia (157 case–control pairs), no statistically significant association was seen with the exposure to UNGD activities within 5 miles or closer to UNGD sites (Table 3). There were eight cases of leukemia and one matched control who lived within less than 0.5 miles away from any UNGD site, which yielded an OR of 7.69 (95% CI 0.70–83.81) after the adjustment for multiple covariates, although it was not statistically significant. We also conducted a subgroup analysis for ALL only (107 case–control pairs) (Table S2). Similarly, no significant association for ALL risk was observed with UNGD exposure measures. Six ALL cases and one control lived within 0.5 miles from a UNGD well, resulting in a non-significant OR of 5.77 (95% CI 0.4–79.16). There was no association between any measure of UNGD exposure and the risk of childhood CNS neoplasms among the 193 pairs of cases and matched controls. The ORs (95% CIs) were 1.28 (0.74–2.22) for ever living within five miles of a UNGD well, 1.15 (0.47–2.79) for the highest quartile of overall UNGD exposure, and 1.96 (0.53–7.26) for living within 0.5 miles of a UNGD well. None of these ORs were statistically significant.

Table 4 presents the results of malignant bone tumors, (n = 43) including Ewing family of tumor. Compared with the non-exposed group, the ORs (95% CIs) were 1.01 (0.25, 4.15) for ever exposed, 3.52 (0.30, 40.73) for the third tertile by overall UNGD exposure, and 3.32 (0.42, 26.24) for living within 2 miles of a UNGD site. None of these ORs were statistically significant. 

We conducted a similar analysis on the Ewing family of tumors, a subgroup of malignant bone tumors. The analysis included 20 cases of the Ewing family of tumors and 498 controls in the present study. Compared with the non-exposed group, the ORs (95% CIs) were 1.55 (0.46–5.17) for ever exposed, 1.39 (0.32–5.69) for the higher (above median) overall UNGD exposure, and 1.72 (0.36–8.36) for living within 2 miles of a UNGD site (Table 5).

### 3.2. Exposure to UNGD Activity and the Risk of Childhood Cancer During the Pregnancy Time Period

We repeated the above analyses for all cancer types with exposures to UNGD during the mother’s pregnancy period. Compared with the non-exposed group, children whose mothers ever lived within 5 miles of a UNGD site during their pregnancy time period had an OR of 0.82 (95% CI 0.47–1.41) for the risk of all four cancer types combined (Table S3), 0.91 (95% CI 0.26–3.12) for the risk of lymphoma (Table S4), 0.73 (95% CI 0.25–2.1) for the risk of leukemia (Table S5), 0.85 (95% CI 0.35–2.03) for the risk of CNS neoplasms (Table S6), 0.22 (95% CI 0.01–8.58) for malignant bone tumors, and 0.55 (95% CI 0.10–2.86) for the Ewing family of tumors (Table S7).

### 3.3. Exposure to Other Environmental Risk Sites and the Risk of Childhood Cancer

We examined whether there was an association with the risk of childhood malignancies by proximity to TRI, UMTRA, and Superfund sites. These analyses were adjusted for age at childbirth, maternal education level, maternal smoking, gestational age, and birthweight. Overall, 86.7% of the children diagnosed with any of the four malignancies studied and 84.7% of their matched controls had a birth residence within 5 miles of a TRI site. Living close to a TRI site was not associated with an elevated risk for the four childhood malignancies combined (Table 6). The malignancy-specific analysis revealed no significantly elevated risk of leukemia, lymphoma, or CNS neoplasms associated with the proximity to a TRI site. Children whose mothers lived within 2–5 miles had on OR of 10.51 (95% CI 1.47–75.37, *p* = 0.019) for the risk of malignant bone tumors, but the OR for <2 miles was not statistically significant (OR = 2.28, 95% CI = 0.52–15.43). The test for linear trends was not significant either (*p* = 0.743).

Overall, 10.6% of cases and 8% of controls had a history of residence within 5 miles of an UMTRA site. There was no increased risk in children for the four childhood malignancies combined nor for leukemia, lymphoma, and malignant bone tumors (Table 7). However, the risk of childhood CNS neoplasms was significantly elevated, with an OR of 2.68 (95% CI 1.11–6.44, *p* = 0.028) for children living within 5 miles of an UMTRA site.

The proportions of children who were exposed to a Superfund site within five miles of residence from birth to index date was 8.8% of cases and 7.8% of controls. There was no statistically elevated risk of all four malignancies combined or any of individual cancer types separately. However, the risk of CNS for children living within 5 miles of a Superfund site was elevated (OR = 2.16, 95% CI 0.96–4.86) but it did not reach the statistical significance level (*p* = 0.06) (Table 7).

## 4. Discussion

The present study considered an overall measure of UNGD activity and proximity in relation to the risk of four childhood cancer types that integrated both the distance and duration of every active well within the time period of interest. Children diagnosed with any of the four malignancies included in the study were significantly more likely to live within a half mile of a UNGD site with a dose–response trend for a higher risk of children’s cancer with closer proximity to an UNGD site within 5 miles (P trend = 0.0041). A higher total UNGD activity was associated with an elevated risk of any of the four childhood cancer types, but the trend test was not statistically significant (P trend = 0.092). This positive association was mainly due to the elevated risk of lymphoma associated with higher levels of exposure to UNGD activities (both Ps for the trend <0.02). The present study did not find statistically significant, consistent associations between the measurements of exposure to UNGD and the risk of leukemia, CNS neoplasms, and malignant bone tumors including the Ewing family of tumors.

Our investigation was also the first to show a statistically significantly elevated risk of lymphoma associated with higher levels of exposure to total UNGD activities or close to the UNGD site, with dose–response relationships. McKenzie et al. [22] was the only study that examined the association between hydraulic fracturing and the risk of childhood non-Hodgkin’s lymphoma. However, their analysis only included 50 cases of non-Hodgkin’s lymphoma. The balance of 528 cases were children diagnosed with cancer other than hematologic malignancies. McKenzie et al. [22] did not find a statistically significant association for the risk of non-Hodgkin’s lymphoma with the density of oil and gas development or IDW well counts within 16.1 km (i.e., 10 miles) of the radius. A higher number of UNGD wells was not associated with an elevated risk of non-Hodgkin’s lymphoma, nor proximity to a UNGD site after the adjustment for age, race, gender, socioeconomic status, elevation, and year of diagnosis. Out of the 50 non-Hodgkin’s lymphoma cases, 18 were unexposed and 32 were within 8 km or a five-mile buffer with UNGD activity exposure. ORs (95% CIs) of non-Hodgkin’s lymphoma were 1.5 (0.72–3.3) in the lowest tertile, 0.91 (0.37–2.2) in the medium tertile, and 1.6 (0.77–3.4) in the highest tertile of exposure to UNGD activities. None of their ORs were statistically significant (which could be due to a smaller number of cases). Our findings are different from this previous study that examined one kind of lymphoma. Future studies are warranted to confirm the findings of our study.

In comparison to the Clark study which noted an increased risk of 1.98 times the odds of developing ALL within 2 km, we conducted a subgroup analysis of ALL case–control pairs only (Table S2). An elevated, albeit non-significant risk of ALL was observed for children in our study whose mothers lived within 0.5 miles (OR = 5.77, 95% CI 0.42–79.16), but not for any other measures of exposure to UNGD. We should caution that our study was different in terms of the exposure time period and age at diagnosis from the study by Clark et al. [23]. Clark noted, after adjustments for maternal race and socioeconomic status, the observed odds ratio was no longer significant (OR = 1.74, 95% CI = 0.93–3.27 and OR = 2.35, 95% CI = 0.93–5.95, respectively) for children residing within 2 miles of a UNGD site. McKenzie et al. [22] also found an elevated risk of ALL (OR = 4.6, 95% CI 1.2–18.0) for children with a highest tertile of UNGD well counts (>33 wells) within 1.6 km (1 mile). Our findings are in line with those of these two previous studies, although our study produced a risk estimate with a wider 95% confidence interval because of the small sample size.

Our study demonstrated a statistically significant elevated risk of CNS neoplasm for children who lived within 5 miles of a UMTRA site (OR = 2.68, 95% CI 1.11–6.44) after adjustments for maternal and infant characteristics from birth records. Legacy Management by the US Department of Energy (DOE) manages over 100 sites in the US associated with past radiological and nuclear material production from the Manhattan Project related to the mining and processing of uranium and hence the exposure to low-level radiation contamination [30]. Four of these UMTRA sites are located in Southwestern Pennsylvania, the region of our study. The environmental cleanup has been completed or the treatment systems for groundwater are in place. DOE performs long-term surveillance and monitoring to make certain that remedies continue to protect the public health and the environment. Out of an abundance of caution, however, we included this environmental exposure in our analysis and observed a positive association for CNS tumors. In addition to radiation exposure from UMTRA, fracturing fluids also contain naturally occurring radioactive materials such as radium-226 and -228 [18,19]. Previous studies [25,33,34] also found that the increased use of ionizing radiation modalities such as cranial computed tomography was associated with an increased risk of CNS tumors [35]. Prospective epidemiologic studies are needed to examine the precise carcinogenic effect of the exposure to ionizing radiation.

This study did not find any evidence in support of risk associations of malignant bone tumors, including the Ewing family of tumors associated with the exposure to UNGD activities and other environmental factors. Given the extremely small number of children with malignant bone tumors, particularly Ewing’s family of tumors, additional studies with a larger sample size may be warranted.

Our study considered all forms of lymphoma (52 Hodgkin’s, 22 NHL, 5 Burkitt’s lymphoma, 25 miscellaneous lymphoreticular neoplasm, and five unspecified), and we were able to consider multiple buffer distances and individual hydraulic fracturing phases as well as an overall metric that considered birth residence. In contrast, McKenzie et al. [22] used geocoded addresses at the time of the cancer diagnosis as the only residence.

Lymphoma is more likely to emerge in the presence of infectious stimuli, chemical toxicity, or an immune system that has lost the ability of self-regulation [36]. There are several studies investigating the possible environmental risk factors for lymphoma in children and adults. Some of the environmental risk factors investigated include polychlorinated biphenyls, organophosphate and organochlorine pesticides, benzene, nitrogen dioxide, and in utero exposure to smoking [37]. Many of these chemicals are in the IARC carcinogen list and are also found in hydraulic fracturing fluids. Future studies with biomarkers for the exposure to UNGD activities may clarify the current study’s observed association between hydraulic fracturing and the risk of lymphoma.

The primary exposure of UNGD activity and the proximity to UNGD for this study was based on birth records for both cases and controls. We sought to determine the extent to which the participant cases and their matched controls remained in the same county or the eight-county study region during the study period. We identified the cases addresses from the Cancer Registry at the time of diagnosis. Among the 507 children with childhood cancer, 445 (87.8%) remained in the same county at the time of the cancer diagnosis as those at the time of birth (Table S8). Similarly, among the 219 control children that responded to our survey request, 201 (91.8%) had remained at the same county at the time of the interview as at the time of birth (Table S9). Given a large proportion of cases and controls remained at the same place from birth to the time of the cancer diagnosis (or index date for controls), a change in residence would not impact on our findings.

This study has many strengths. It is the first population-based study on UNGD activities and the childhood cancer risk randomly sampling age-, race-, and sex-matched controls from birth records. The study population was restricted to Southwestern Pennsylvanian counties which have permitted UNGD activities since 2005. As such, the City of Pittsburgh was excluded due to a ban on hydraulic fracturing. This minimized potential confounding and bias due to other environmental risk factors. The rigid matching criteria (less than 45 days of the difference in birth dates between a case and a matched control) eliminated the potential confounding effect by age. The collection of other environmental exposure data through publicly available sources provided additional information on factors (e.g., TRI, UMTRA, and Superfund sites), which were adjusted for through multivariable logistic models.

In contrast to other studies that used well counts and IDW well counts as exposure variables, our study team was able to create a new metric called “overall UNGD exposure” to evaluate the cancer risk. The challenge in considering the health effects of individual hydraulic fracturing phases particularly with a condition such as cancer with a longer latency period, is that they may be occurring simultaneously in the background with other co-located wells. This overall UNGD exposure metric simultaneously accounted for the duration of UNGD activity (including all four phases, well pad preparation, drilling, fracturing, and production) and the distance of the wells from the residential address at the time of birth. Wells closer to the residence of birth are given a greater weight than those further away. Moreover, other potential environmental covariates including the proximity to TRI, UMTRA, and Superfund sites were included in the overall analysis. An additional strength was the application of multiple buffers for the proximity of residences within <0.5, 0.5–1.0, 1–2, and 2–5 miles of these sites, which allowed for the assessment of the cancer risk with UNGD proximity. The increased risk of childhood cancer with decreasing residential distance from UNGD sites suggests a probable link between UNGD activities and the childhood cancer risk.

This comprehensive analysis also revealed consistent associations for various metrics of UNGD activities, which were highly correlated with each other and the risk of childhood cancer outcomes, further strengthening a probable link between UNGD activities in general and the risk of childhood cancer.

Our study is also the first study to include the four most common childhood cancers—leukemia, lymphoma, CNS neoplasms, and malignant bone tumors. The inclusion of multiple cancer types provided a larger sample size for the study and allowed for the assessment of cancer-specific risks with UNGD activities. The strongest association was observed between UNGD activities and the risk of childhood lymphoma, which are novel findings and warrant assessments by future studies.

The present study also has some limitations. Whereas our overall UNGD exposure metric has a duration of time, proximity, and density components, it may be affected by many factors such as the nearby topography and geological formations, weather patterns, and water sources, and the behaviors of individuals residing near UNGD activity. It is possible that using the closest well proximity distance as a proxy for exposure has resulted in misclassification bias, which may identify an association where there is not one or vice versa. Although there was an attempt to include UNGD open-source data from Ohio and West Virginia, that data was reported only as annual estimates. In addition, we used the residence from the birth records as a proxy for UNGD exposure from birth until index date which also introduces the possibility of misclassification bias. However, there was a high concordance (88–92%) of residences at birth compared to their residence at diagnosis (cases) or birth compared to their residence at index date (controls) remaining in the same county. This adds validity to the use of birth certificates as a proxy for UNGD metrics for this study. Another limitation of the study was the small sample size particularly for bone cancer and the Ewing Family of Tumors which resulted in large variations in risk estimates and wider confidence intervals.

## 5. Conclusions

The present study combined a spatial measure of proximity to Unconventional Natural Gas Development well activity with a temporal measure (days) of well activity to create an overall cumulative UNGD exposure with which to consider the childhood cancer risk. We considered the four most common childhood cancers (CNS, leukemia, lymphoma, and bone malignant tumors) that have been linked in the literature to environmental exposures and showed a significant increased risk of lymphoma and all four types of childhood cancers combined among children residing less than 1 mile from their birth residence compared to a similar group of healthy children. These findings are generally consistent with results from the previous two childhood cancer studies. Together these results suggest additional studies are warranted with larger sample sizes and measurements of more specific chemical compounds in the water and soil near fracking installations. The observed elevated risk of lymphoma in children living in close proximity to a UNGD site provides evidence in support of the development of a public health policy that would safeguard residents from living too close to a UNGD installation.

## Figures and Tables

**Table 1:** Characteristics of children with childhood cancer.

Characteristic	Sub-Characteristic	Cases, n (%)
Total number		498
Cancer type	Leukemias, myeloproliferative diseases, and myelodysplastic diseases	157 (31.5)
	Lymphomas and reticuloendothelial neoplasms	105 (21.1)
	Central nervous system and miscellaneous intracranial and intraspinal neoplasms	193 (38.8)
	Malignant bone tumors including Ewing family of tumors	43 (8.6)
Age at diagnosis (years)	0–4	145 (29.1)
	5–9	95 (19.1)
	10–14	109 (21.9)
	15–19	146 (29.3)
	20–24 (Ewing cancer only)	2 (0.4)
	25–29 (Ewing cancer only)	1 (0.2)
Year at diagnosis of childhood cancer	2010	59 (11.9)
	2011	63 (12.7)
	2012	44 (8.8)
	2013	52 (10.4)
	2014	46 (9.2)
	2015	50 (10.0)
	2016	52 (10.4)
	2017	40 (8.0)
	2018	48 (9.6)
	2019	44 (8.8)

**Table 2:** Distributions of sociodemographic and birth characteristics of mothers and children with and without childhood cancer.

Characteristics	Sub-Characteristic	Cases (%)	Controls (%)
Total subjects		498	498
Sex	Female	216 (43.4)	216 (43.4)
	Male	282 (56.6)	282 (56.6)
Birth Year	1990–1994	46 (9.2)	47 (9.4)
	1995–1999	107 (21.5)	104 (20.9)
	2000–2004	111 (22.3)	112 (22.5)
	2005–2009	104 (20.9)	106 (21.3)
	2010–2014	94 (18.9)	94 (18.9)
	2015–2018	36 (7.2)	35 (7.0)
County of Residence	Allegheny (excluding City of Pittsburgh)	201 (40.4)	201 (40.4)
	Armstrong	14 (2.8)	14 (2.8)
	Beaver	31 (6.2)	31 (6.2)
	Butler	68 (13.7)	68 (13.7)
	Fayette	25 (5.0)	25 (5.0)
	Greene	12 (2.4)	12 (2.4)
	Washington	60 (12.0)	60 (12.0)
	Westmoreland	87 (17.5)	87 (17.5)
Birthweight	≤2500 g	28 (5.4)	23 (4.6)
	2501–4000 g	411 (82.5)	426 (85.5)
	>4000 g	60 (12.1)	49 (9.8)
Number of Prenatal Visits	0–7	41 (8.2)	48 (9.6)
	8–12	241 (48.4)	245 (49.2)
	13–16	177 (35.5)	176 (35.3)
	≥17	20 (4.0)	17 (3.4)
	Unknown	19 (3.8)	12 (2.4)
Maternal Age (years)	<20	33 (6.6)	25 (5.0)
	20–24	79 (15.9)	83 (16.7)
	25–29	132 (26.5)	124 (24.9)
	30–34	146 (29.3)	160 (32.1)
	≥35	108 (21.7)	106 (21.3)
Maternal Race	White	480 (96.4)	480 (96.4)
	Black	12 (2.4)	12 (2.4)
	Other	5 (1.0)	6 (1.2)
	Unknown/Refused	1 (0.2)	0 (0.0)
Maternal Education	≤8th Grade	2 (0.4)	3 (0.6)
	Some High School	36 (7.2)	25 (5.0)
	High School Diploma	145 (29.1)	141 (28.3)
	Some College	124 (24.9)	123 (24.7)
	College Degree or Higher	186 (37.4)	198 (39.8)
	Unknown	5 (1.0)	8 (1.6)
Maternal Smoking During Pregnancy	Never	397 (79.7)	408 (81.9)
	Ever	92 (18.5)	89 (17.9)
	Unknown	9 (1.8)	1 (0.2)
Gestation in Weeks	Mean (S.D.)	38.7 (1.8)	38.8 (1.6)

**Table 3:** Odds ratio (OR) of lymphoma, leukemia, CNS neoplasms, and combined childhood malignancies by the overall UNGD exposure and by proximity to the closest active well from birth to diagnosis (index) date.

Risk Measures of Overall UNGD Exposure ^1^ and Proximity	All 4 Cancers Combined ^2^			Lymphoma			Leukemia			CNS Neoplasms		
	Cases	Ctrls ^3^	OR (95% CI)	Cases	Ctrls	OR (95%CI)	Cases	Ctrls	OR (95%CI)	Cases	Ctrls	OR (95%CI)
By ever-exposed within 5 miles												
Non-exposed	187	201	1.00 (reference)	32	40	1.00 (reference)	69	67	1.00 (reference)	74	83	1.00 (reference)
Exposed	311	297	1.24 (0.87, 1.78)	73	65	2.24 (0.92, 5.47)	88	90	0.79 (0.35, 1.79)	119	110	1.28 (0.74, 2.22)
By overall UNGD exposure within 5 miles												
Non-exposed	187	201	1.00 (reference)	32	40	1.00 (reference)	69	67	1.00 (reference)	74	83	1.00 (reference)
1st quartile	86	74	1.40 (0.91, 2.14)	15	13	1.74 (0.53, 5.77)	31	25	1.16 (0.46, 2.90)	34	29	1.32 (0.69, 2.50)
2nd quartile	50	74	0.76 (0.46, 1.25)	11	18	1.14 (0.35, 3.72)	9	23	0.38 (0.13, 1.16)	24	24	1.06 (0.48, 2.33)
3rd quartile	88	74	1.69 (1.01, 2.82)	24	15	5.68 (1.58, 20.48)	25	26	0.98 (0.29, 3.27)	30	24	1.55 (0.71, 3.35)
4th quartile	87	75	1.79 (1.00, 3.19)	23	19	3.96 (1.01, 15.49)	23	16	1.51 (0.35, 6.42)	31	33	1.15 (0.47, 2.79)
*p* trend			0.0975			0.0155			0.7676			0.6205
By buffer zone (proximity to closest well to case/control residence)												
Non-exposed	187	201	1.00 (reference)	32	40	1.00 (reference)	69	67	1.00 (reference)	74	83	1.00 (reference)
(2–5] miles	170	178	1.18 (0.82, 1.71)	39	39	2.06 (0.83, 5.13)	50	56	0.77 (0.34, 1.75)	62	62	1.23 (0.71, 2.16)
(1–2] miles	77	72	1.49 (0.89, 2.51)	16	17	2.45 (0.77, 7.83)	20	21	0.97 (0.28, 3.33)	30	28	1.54 (0.69, 3.47)
(0.5–1] miles	38	37	1.61 (0.85, 3.03)	12	6	5.05 (1.09, 23.39)	10	12	0.92 (0.24, 3.46)	15	15	1.38 (0.49, 3.89)
[0–0.5] miles	26	10	3.94 (1.66, 9.39)	6	3	7.71 (1.01, 59.00)	8	1	7.69 (0.70, 83.91)	8	5	1.96 (0.53, 7.26)
*p* trend			*p* = 0.0041			0.0149			0.3203			0.2818

^1^ Adjusted for maternal age at childbirth, maternal education level, maternal smoking status at childbirth, gestation age, birthweight, TRI, UMTRA, and Superfund ^2^ See Table 4 for malignant bone tumors. ^3^ Controls shown in Table 4 are the results for malignant tumors of the bone. No statistically significant association was observed with any exposure measurements of UNGD activity. Higher ORs were observed with the highest tertile of the total UNGD activities (OR = 3.52, 95% CI 0.3–40.73) or living within 2 miles of a UNGD site (OR = 3.32, 95% CI 0.42–26.24). None of the ORs were statistically significant.

**Table 4:** Odds ratio (OR) of malignant bone tumors by overall UNGD exposure and by proximity to the closest active well from the birth to diagnosis (index) date.

Risk Measures of Overall UNGD Exposure and Proximity	Exposure Groups	Cases	Controls	OR (95% CI) *
By ever-exposed within 5 miles	Non-exposed	12	11	1.00 (reference)
	Exposed	31	32	1.01 (0.25, 4.15)
By overall UNGD exposure within 5 miles	Non-exposed	12	11	1.00 (reference)
	1st tertile	9	11	1.20 (0.25, 5.85)
	2nd tertile	9	12	0.63 (0.1, 4.03)
	3rd tertile	13	9	3.52 (0.30, 40.73)
	*p* trend			0.5410
By buffer zone (proximity to closest well to case/control residence)	Non-exposed	12	11	1.00 (reference)
	(2–5] miles	15	21	1.02 (0.25, 4.12)
	[0–2] miles	16	11	3.32 (0.42, 26.24)
	*p* trend			0.2550

* Adjusted for maternal age at childbirth, maternal education level, maternal smoking status at childbirth, gestation age, birthweight, TRI, UMTRA, and Superfund.

**Table 5:** Odds ratio of the Ewing family of tumors by the overall UNGD exposure and by proximity to the closest active well from the birth to diagnosis (index) date.

Risk Measures of Overall UNGD Exposure and Proximity	Exposure Groups	Cases	Controls	OR (95% CI) *
By ever-exposed within 5 miles	Non-exposed	6	201	1.00 (reference)
	Exposed	14	297	1.55 (0.46, 5.17)
By overall UNGD exposure within 5 miles	Non-exposed	6	201	1.00 (reference)
	Below Median	8	148	1.62 (0.46, 5.7)
	Above Median	6	149	1.39 (0.32, 5.96)
	*p* trend			0.6763
By buffer zone (proximity to closest well to case/control residence)	Non-exposed	6	201	1.00 (reference)
	(2–5] miles	9	178	1.50 (0.43, 5.21)
	[0–2] miles	5	119	1.72 (0.36, 8.36)
	*p* trend			0.4879

* Derived from unconditional logistic regressions including total controls with the adjustments for matching factors (date of birth, age, sex, race, and county of residence), maternal age at childbirth, maternal education level, maternal smoking status at childbirth, gestation age, birthweight, TRI, UMTRA, and Superfund sites.

**Table 6:** Odds ratio of childhood malignancies by proximity to the closest toxic release inventory (TRI) sites from the birth to diagnosis (index) date.

Cancer	Measures of Exposure to TRI Sites	Cases	Controls	OR (95% CI) *	*p* Value	*P* for Trend
All four cancers combined	Non-exposed	66	76	1 (reference)	-	0.54
	(2–5] miles	197	194	1.23 (0.81, 1.86)	0.34	
	(1–2] miles	132	125	1.27 (0.8, 2.01)	0.32	
	(0.5–1] miles	69	72	1.15 (0.69, 1.92)	0.58	
	[0–0.5] miles	34	31	1.31 (0.71, 2.42)	0.39	
Leukemia	Non-exposed	19	20	1 (reference)	-	0.32
	(2–5] miles	61	64	1.23 (0.55, 2.74)	0.62	
	(1–2] miles	43	46	1.12 (0.48, 2.63)	0.79	
	(0.5–1] miles	24	17	1.86 (0.68, 5.05)	0.23	
	[0–0.5] miles	10	10	1.61 (0.47, 5.55)	0.45	
Lymphoma	Non-exposed	15	16	1 (reference)	-	0.39
	(2–5] miles	36	38	1.14 (0.37, 3.44)	0.82	
	(1–2] miles	34	30	1.45 (0.46, 4.51)	0.52	
	(0.5–1] miles	10	17	0.59 (0.14, 2.51)	0.47	
	[0–0.5] miles	10	4	3.89 (0.71–21.41)	0.12	
CNS Neoplasms	Non-exposed	29	29	1 (reference)	-	0.86
	(2–5] miles	78	82	0.99 (0.52, 1.91)	0.98	
	(1–2] miles	44	40	1.16 (0.54, 2.46)	0.71	
	(0.5–1] miles	31	29	1.11 (0.51–2.4)	0.80	
	[0–0.5] miles	11	13	0.92 (0.36, 2.34)	0.86	
Malignant Bone Tumors	Non-exposed	3	11	1 (reference)	-	0.73
	(2–5] miles	22	10	10.51 (1.47, 75.37)	0.019	
	[0–2] miles	18	22	2.82 (0.52, 15.43)	0.23	

* Adjusted for maternal age at childbirth, maternal education level, maternal smoking status at childbirth, gestation age, and birthweight.

**Table 7:** Odds ratio of childhood malignancies by living within five miles from a uranium mill tailing remedial action (UMTRA) or Superfund site from the birth to diagnosis (index) date.

		Cancer Risk by Exposure to UMTRA				Cancer Risk by Exposure to Superfund			
Cancer	Exposure Category	Cases	Controls	OR (95% CI) *	*p* Value	Cases	Controls	OR (95% CI) *	*p* Value
All four cancers combined	Non-exposed	445	456	1 (reference)	-	454	459	1 (reference)	-
	[0–5] miles	53	42	1.37 (0.86, 2.2)	0.19	44	39	1.12 (0.71, 1.76)	0.64
Leukemia	Non-exposed	140	140	1 (reference)	-	142	139	1 (reference)	-
	[0–5] miles	17	17	0.95 (0.37, 2.43)	0.91	15	18	0.64 (0.29, 1.41)	0.27
Lymphoma	Non-exposed	97	95	1 (reference)	-	99	97	1 (reference)	-
	[0–5] miles	8	10	0.75 (0.25, 2.2)	0.60	6	8	0.82 (0.28, 2.4)	0.71
CNS Neoplasms	Non-exposed	97	95	1 (reference)	-	99	97	1 (reference)	-
	[0–5] miles	8	10	0.75 (0.25, 2.2)	0.60	6	8	0.82 (0.28, 2.4)	0.71
Malignant bone tumors	Non-exposed	36	37	1 (reference)	-	41	41	1 (reference)	-
	[0–5] miles	7	6	1.40 (0.38, 5.13)	0.62	2	2	0.77 (0.1, 6.01)	0.81

* Adjusted for maternal age at childbirth, maternal education level, maternal smoking status at childbirth, gestation age, and birthweight.

## Data Availability

Data for wells was available from open-source data described in the paper and likewise the data specific to the study population would require a protocol request and applications to the Bureau of Health Statistics at the Pennsylvania Department of Health.

## Supplementary Materials

The following supporting information can be downloaded at https://www.mdpi.com/article/10.3390/ijerph22010068/s1; Table S1: Definition of Childhood Cancer Cases for the Case–Control Study in Southwestern PA (International Classification of Childhood Cancer Recode Third Edition, ICD-O-3/IARC 2017); Table S2: Odds Ratio of Acute Lymphocytic Leukemia (ALL) by Overall UNGD Exposure and by Proximity to Closest Active Well Birth to Diagnosis (Index) Date Table S3: Odds Ratio of Combined Childhood Malignancies by Overall UNGD Exposure and by Proximity to Closest Active Well During Pregnancy Time Period; Table S4: Odds Ratio of Lymphoma by Overall UNGD Exposure and by Proximity to Closest Active Well During Pregnancy Time Period; Table S5: Odds Ratio of Leukemia by Overall UNGD Exposure and by Proximity to Closest Active Well During Pregnancy Time Period; Table S6: Odds Ratio of Central Nervous System (CNS) Neoplasms by Overall UNGD Exposure and by Proximity to Closest Active Well During Pregnancy Time Period; Table S7: Odds Ratio of Malignant Bone Tumors and Ewing Family of Tumors by Overall UNGD Exposure Well During Pregnancy Time Period; Table S8: County of the mother’s residence when giving birth, vs. County at diagnosis for the 507 Childhood Cancers identified for the Study; Table S9: County of the mother’s residence when giving birth vs. county at index date for 219 matched controls surveyed in the study period (2020–2022); Figure S1: Map of Uranium Mill Tailing Remedial Action (UMTRA) Sites in Southwestern Pennsylvania and Surrounding States and Counties; Figure S2: Map of Superfund Sites in Southwestern Pennsylvania and Surrounding States and Counties; Figure S3: Map of Toxic Release Inventory (TRI) Sites in Southwestern Pennsylvania and Surrounding States and Counties.
