# Supplementary material for: Cumulative Exposure to Unconventional Natural Gas Development and the Risk of Childhood Cancer: A Registry-Based Case–Control Study
Source: Int J Environ Res Public Health. 2025 Jan 7;22(1):68. doi: 10.3390/ijerph22010068 (PMC11764582; doi:10.3390/ijerph22010068)

Supplementary Tables

Table S1. Definition of Childhood Cancer Cases for the Case-Control Study in Southwestern PA (International Classification of Childhood Cancer Recode Third Edition, ICD-O-3/IARC 2017)

Cancer type	ICCC Recode 3rd ICD-O-3/ IARC 2017 morphology codes	Behavior codes	ICD-O-3 primary site code
**I. Leukemias, Myeloproliferative, and Myelodysplastic Diseases (0-19 years of age)**			
1. Precursor cell leukemia	9811-9818, 9837	3	C420, C421, C423, C424, C809
	9835, 9836	3	C000-C809
2.Mature B-cell leukemias	9823	3	C420, C421, C423, C424, C809
	9826, 9832, 9833, 9940	3	C000-C809
3. Mature T-cell and Natural Killer (NK) cell leukemias	9827	3	C420, C421, C423, C424, C809
	9831, 9834, 9948	3	C000-C809
4. Lymphoid leukemia, NOS	9591	3	C420, C421, C423, C424
	9820	3	C000-C809
5. Acute myeloid leukemias	9840, 9861, 9865-9867, 9869-9874, 9891, 9895-9897, 9898, 9910, 9911, 9920, 9930, 9931	3	C000-C809
6. Chronic myeloproliferative diseases	9863, 9875, 9876, 9950, 9960-9964	3	C000-C809
7. Myelodysplastic syndrome and other myeloproliferative diseases	9945, 9946, 9975, 9980, 9982-9987, 9989, 9991, 9992	3	C000-C809
8. Unspecified and other specified leukemias	9800, 9801, 9805-9809, 9860, 9965-9967	3	C000-C809
**II. Lymphoma (0-19 years of age)**			
1. Precursor cell lymphomas	9727-9729	3	C000-C809
	9811-9818, 9837	3	C000-C419, C422, C440-C779
2. Mature B-cell lymphomas (except Burkitt lymphoma)	9597, 9670, 9671, 9673, 9675, 9678-9680, 9684, 9688-9691, 9695, 9698, 9699, 9712, 9731-9735, 9737, 9738, 9761, 9762, 9764-9766, 9769, 9970, 9971	3	C000-C809
	9823	3	C000-C419, C422, C440-C779
3. Mature T-cell and NK-cell lymphomas	9700-9702, 9705, 9708, 9709, 9714, 9716-9719, 9724-9726, 9767, 9768	3	C000-C809
	9827	3	C000-C419, C422, C440-C779
4. non-Hodgkin lymphomas, NOS	9591	3	C000-C419, C422, C440-C779, C809
	9760	3	C000-C809
5. Burkitt lymphoma	9687	3	C000-C809
6. Miscellaneous lymphoreticular neoplasms	9740-9742, 9750, 9751, 9754-9759	3	C000-C809
7. Unspecified lymphomas	9590, 9596	3	C000-C809
**III. CNS and Miscellaneous Intracranial and Intraspinal Neoplasms (0-19 years of age)**			

1. Ependymomas and choroid plexus tumor	9383, 9390, 9391-9394, 9396	0-1, 3	C000-C809
2. Astrocytomas	9380	0-1, 3	C723
	9384, 9400-9411, 9420-9424, 9425, 9440-9442	0-1, 3	C000-C809
3. Intracranial and intraspinal embryonal tumors	9470-9478, 9480, 9508	0-1, 3	C000-C809
	9501-9504	0-1, 3	C700-C729
4. Other gliomas	9381, 9382, 9385, 9430, 9431, 9444, 9445, 9450, 9451, 9460	0-1, 3	C000-C809
	9380	0-1, 3	C700-C722, C724-C729, C751, C753
5. Other specified intracranial and intraspinal neoplasms	9840, 9861, 9865-9867, 9869-9874, 9891, 9895-9897, 9898, 9910, 9911, 9920, 9930, 9931	3	C000-C809
	8158, 8290	0-1, 3	C751
6. Unspecified intracranial and intraspinal neoplasms	8000-8005	0-1, 3	C700-C729, C751-C753
**IV. Malignant Bone Tumor (0-29 years)**			
1. Osteosarcoma	9180–9187, 9191–9195, 9200	3	C400-C419, C760-C768, C809
2. Chondrosarcomas	9210, 9220, 9240	3	C400-C419, C760-C768, C809
	9211-9213, 9221, 9222, 9230, 9241-9243		C000-C809
	9231		C400-C419
3. Ewing tumor and related sarcomas of bone	9260	3	C400-C419, C760-C768, C809
	9365		C000-C809
	9364		C000-C809
4.. Other specified malignant bone tumors	8810, 8811, 8818, 8823, 8830	3	C400-C419
	8812, 9262, 9370-9372, 9270-9275, 9280-9282, 9290, 9300-9302, 9310-9312, 9320-9322, 9330, 9340-9342, 9250, 9261		C000-C809
5. Unspecified malignant bone tumors	8000-8005, 8800, 8801, 8803-8805	3	C400-C419

Table S2. Odds Ratio of Acute Lymphocytic Leukemia (ALL) by Overall UNGD Exposure and by Proximity to Closest Active Well Birth to Diagnosis (Index) Date

**Overall UNGD Exposure and Proximity**	**ALL**		
	**Cases**	**Controls**	**OR (95% CI)**
By ever exposed within 5 miles			
Non-exposed	45	42	1.00 (reference)
Exposed	62	65	0.57 (0.18, 1.77)
By overall UNGD exposure within 5 miles			
Non-exposed	45	42	1.00 (reference)
1st quartile	19	16	0.84 (0.24, 2.87)
2nd quartile	8	16	0.30 (0.07, 1.27)
3rd quartile	12	16	0.50 (0.1, 2.44)
4th quartile	23	17	0.87 (0.16, 4.78)
P trend			*0.6439*
By buffer zone (proximity to closest well to case/control residence)			
Non-exposed	45	42	1.00 (reference)
(2-5] mi	32	37	0.55 (0.17, 1.74)
(1-2] mi	15	17	0.73 (0.14, 3.79)
(0.5-1] mi	9	10	0.72 (0.15, 3.55)
[0-0.5] mi	6	1	5.77 (0.42, 79.16)
P trend			*0.4010*

Adjusted for maternal age at childbirth, maternal education level, maternal smoking status at childbirth, gestation age, birthweight, TRI, UMTRA, and Superfund site

Table S3. Odds Ratio of Combined Childhood Malignancies by Overall UNGD Exposure and by Proximity to Closest Active Well During Pregnancy Time Period

**Overall UNGD Exposure and Proximity**	**All 4 Cancers Combined**		
	**Cases**	**Controls**	**OR (95% CI)**
By ever exposed within 5 miles			
Non-exposed	404	399	1.00
Exposed	94	99	0.82 (0.47, 1.41)
By overall UNGD exposure within 5 miles			
Non-exposed	404	399	1.00
1st quartile	17	24	0.63 (0.29, 1.34)
2nd quartile	22	25	0.77 (0.37, 1.64)
3rd quartile	36	25	1.40 (0.63, 3.14)
4th quartile	19	25	0.75 (0.31, 1.83)
P trend			*0.7587*
By buffer zone (proximity to closest well to case/control residence)			
Non-exposed	404	399	1.00
(2-5] mi	63	64	0.84 (0.48, 1.46)
(1-2] mi	22	24	0.72 (0.31, 1.67)
(0.5-1] mi	7	9	0.65 (0.19, 2.26)
[0-0.5] mi	2	2	0.81 (0.05, 14.62)
P trend			*0.3817*

Adjusted for maternal age at childbirth, maternal education level, maternal smoking status at childbirth, gestation age, birthweight, TRI, UMTRA, and Superfund site

Table S4. Odds Ratio of Lymphoma by Overall UNGD Exposure and by Proximity to Closest Active Well During Pregnancy Time Period

**Overall UNGD Exposure and Proximity**	**Lymphoma**		
	**Cases**	**Controls**	**OR (95% CI)**
By ever exposed within 5 miles			
Non-exposed	90	89	1.00
Exposed	15	16	0.91 (0.26, 3.12)
By overall UNGD exposure within 5 miles			
Non-exposed	90	89	1.00
1st quartile	1	5	0.28 (0.03, 2.60)
2nd quartile	5	5	0.82 (0.13, 5.06)
3rd quartile	6	3	4.83 (0.4, 58.83)
4th quartile	3	3	3.59 (0.25, 50.69)
P trend			*0.4023*
By buffer zone (proximity to closest well to case/control residence)			
Non-exposed	90	89	1.00
(2-5] mi	9	10	0.96 (0.27, 3.48)
(1-2] mi	2	3	0.77 (0.09, 6.34)
(0.5-1] mi	2	1	1.82 (0.11, 30.83)
[0-0.5] mi	2	2	2.26 (0.06, 85.26)
P trend			*0.6818*

Adjusted for maternal age at childbirth, maternal education level, maternal smoking status at childbirth, gestation age, birthweight, TRI, UMTRA, and Superfund site

Table S5. Odds Ratio of Leukemia by Overall UNGD Exposure and by Proximity to Closest Active Well During Pregnancy Time Period

**Overall UNGD Exposure and Proximity**	**Leukemia**		
	**Cases**	**Controls**	**OR (95% CI)**
By ever exposed within 5 miles			
Non-exposed	122	120	1.00
Exposed	35	37	0.73 (0.25, 2.10)
By overall UNGD exposure within 5 miles			
Non-exposed	122	120	1.00
1st quartile	8	8	0.89 (0.24, 3.27)
2nd quartile	6	10	0.44 (0.10, 1.90)
3rd quartile	14	9	1.12 (0.24, 5.25)
4th quartile	7	10	0.47 (0.08, 2.64)
P trend			*0.4337*
By buffer zone (proximity to closest well to case/control residence)			
Non-exposed	122	120	1.00
(2-5] miles	25	21	0.77 (0.27, 2.24)
[0-2] miles	10	16	0.27 (0.05, 1.36)
P trend			*0.1288*

Adjusted for maternal age at childbirth, maternal education level, maternal smoking status at childbirth, gestation age, birthweight, TRI, UMTRA, and Superfund site

Table S6. Odds Ratio of Central Nervous System (CNS) Neoplasms by Overall UNGD Exposure and by Proximity to Closest Active Well During Pregnancy Time Period

**Overall UNGD Exposure and Proximity**	**CNS Neoplasms**		
	**Cases**	**Controls**	**OR (95% CI)**
By ever exposed within 5 miles			
Non-exposed	152	151	1.00
Exposed	41	42	0.85 (0.35, 2.03)
By overall UNGD exposure within 5 miles			
Non-exposed	152	151	1.00
1st quartile	8	9	0.77 (0.18, 3.30)
2nd quartile	10	10	0.99 (0.28, 3.47)
3rd quartile	14	11	1.09 (0.34, 3.53)
4th quartile	9	12	0.56 (0.15, 2.03)
P trend			*0.5827*
By buffer zone (proximity to closest well to case/control residence)			
Non-exposed	152	151	1.00
(2-5] miles	28	29	0.84 (0.34, 2.06)
(1-2] miles	8	7	1.07 (0.26, 4.46)
[0-1] miles	5	6	0.68 (0.13, 3.59)
P trend			*0.7712*

Adjusted for maternal age at childbirth, maternal education level, maternal smoking status at childbirth, gestation age, birthweight, TRI, UMTRA, and Superfund site

Table S7. Odds Ratio of Malignant Bone Tumors and Ewing Family of Tumors by Overall UNGD Exposure Well During Pregnancy Time Period

**Overall UNGD Exposure**	**Malignant Bone Tumors**			**Ewing Family of Tumors**		
	**Cases**	**Controls**	**OR (95% CI)**	**Cases**	**Controls**	**OR (95% CI)**
By ever exposed within 5 miles						
Non-exposed	40	39	1.00	18	399	1.00
Exposed	3	4	0.22 (0.01, 8.58)	2	99	0.55 (0.10, 2.86)

Adjusted for maternal age at childbirth, maternal education level, maternal smoking status at childbirth, gestation age, birthweight, TRI, UMTRA, and Superfund site

Table S8. County of the mother's residence when giving birth, vs. County at diagnosis for the 507 Childhood Cancers identified for the Study

Child's Birth County	Child's Diagnosis County									
	Allegheny	Armstrong	Beaver	Butler	Fayette	Greene	Washington	Westmoreland	Total	%
Allegheny	188	0	1	8	1	0	6	9	213	88.3
Armstrong	0	13	0	0	0	0	0	3	16	81.3
Beaver	1	1	30	3	0	0	0	0	37	81.1
Butler	0	0	1	55	0	0	0	0	58	94.8
Fayette	2	0	0	0	23	1	2	1	29	79.3
Greene	0	0	0	0	0	9	3	0	12	75.0
Washington	4	0	0	0	0	2	49	0	55	89.1
Westmoreland	7	0	0	0	1	0	1	78	87	89.7
Total	204	14	32	68	25	12	61	91	507*	

*9 cases were excluded in the analysis due to mismatching

Table S9. County of the mother's residence when giving birth vs county at index date for 219 matched controls surveyed in the study period (2020-2022)

Child's Birth County	Child's Index Date County									
	Allegheny	Armstrong	Beaver	Butler	Fayette	Greene	Washington	Westmoreland	Total	%
Allegheny	92	0	1	1	0	0	4	1	99	92.9
Armstrong	0	4	0	0	0	0	0	0	4	100
Beaver	2	0	14	2	0	0	0	0	18	77.8
Butler	2	0	0	16	0	0	0	0	18	88.9
Fayette	0	0	0	0	6	0	0	1	7	85.7
Greene	0	0	0	0	0	6	1	0	7	85.7
Washington	1	0	0	1	0	0	24	0	26	92.3
Westmoreland	0	1	0	0	0	0	0	39	40	97.5
Total	97	5	15	20	6	6	29	41	219	

Figure S1. Map of Uranium Mill Tailing Remedial Action (UMTRA) Sites in Southwestern Pennsylvania and Surrounding States and Counties

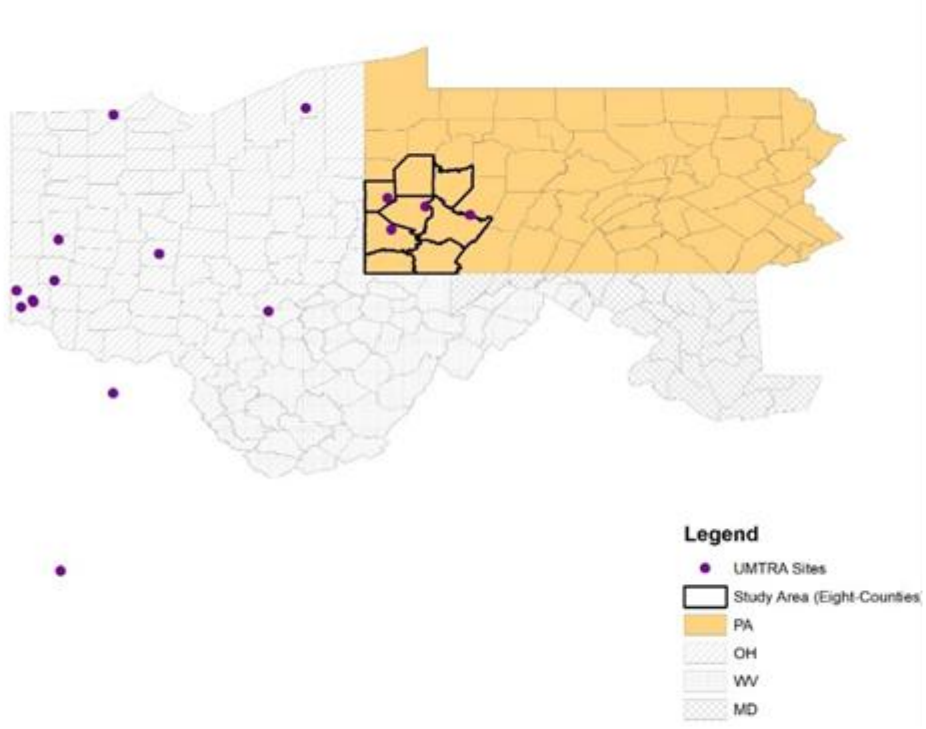

Figure S2. Map of Superfund Sites in Southwestern Pennsylvania and Surrounding States and Counties

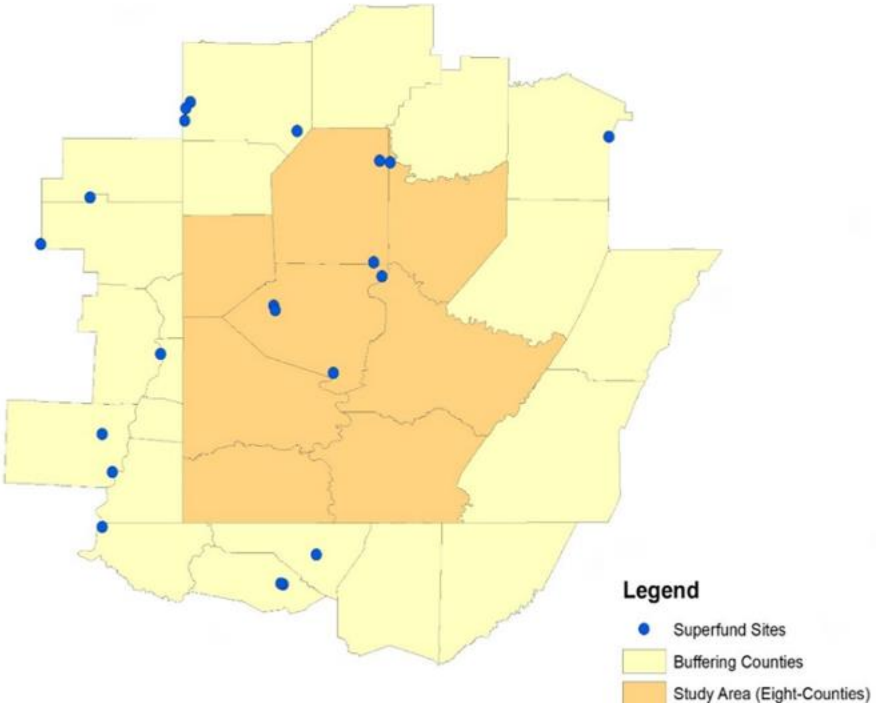

Figure S3. Map of Toxic Release Inventory (TRI) Sites in Southwestern Pennsylvania and Surrounding States and Counties

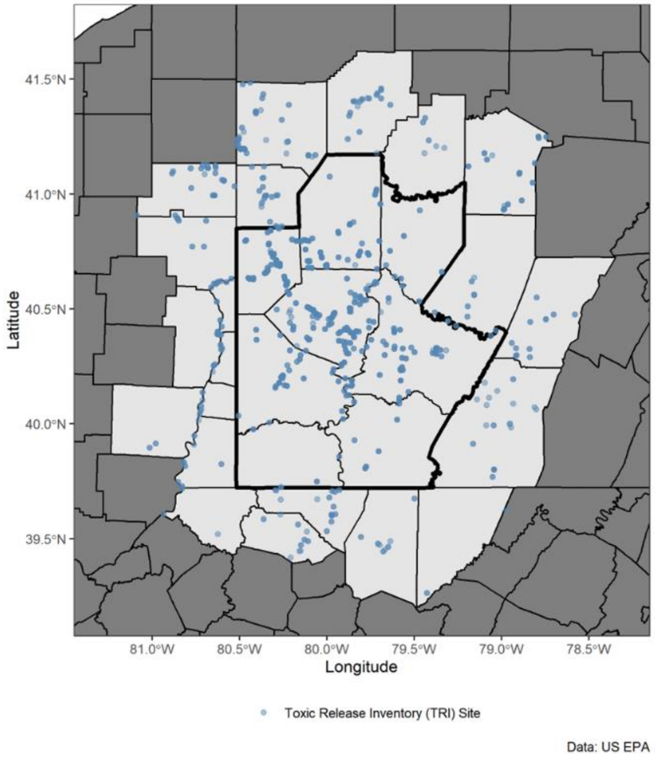

Supplement: Supplementary file 1 [file ijerph-22-00068-s001.zip › ijerph-3341939-supplementary.pdf]
